# Supplementary material for: Antiferromagnetic Spin Wave Field-Effect Transistor
Source: Sci Rep. 2016 Apr 6;6:24223. doi: 10.1038/srep24223 (PMC4822171; doi:10.1038/srep24223)
Supplement: Supplementary Information [file srep24223-s1.pdf]

Supplementary Materials for "Antiferromagnetic Spin Wave Field-Effect Transistor"

Ran Cheng,[1] Matthew W. Daniels,[1] Jian-Gang Zhu,[2] and Di Xiao[1]

[1]*Department of Physics, Carnegie Mellon University, 5000 Forbes Avenue, Pittsburgh, PA 15213*
[2]*Department of Electrical and Computer Engineering, Carnegie Mellon University, 5000 Forbes Avenue, Pittsburgh, PA 15213*

S1. DZYALOSHINSKII-MORIYA INTERACTION

On a two dimensional bipartite lattice with mirror symmetry breaking along $\hat{\boldsymbol{z}}$, the nearest neighboring DMI is

$$H_D = \sum_{<ij>} \boldsymbol{D}_{ij} \cdot \boldsymbol{S}_i \times \boldsymbol{S}_j = -D \sum_i \boldsymbol{S}_i \cdot \left\{ \sum_{<j>} (\hat{\boldsymbol{z}} \times \hat{\boldsymbol{r}}_{ij}) \times \boldsymbol{S}_j \right\}. \tag{S1}$$

If we assume that spins on the i-sites are the A-sublattice and consider that $\boldsymbol{m} = (\boldsymbol{S}_A + \boldsymbol{S}_B)/2S$ and $\boldsymbol{n} = (\boldsymbol{S}_A - \boldsymbol{S}_B)/2S$, then $\boldsymbol{S}_i = S(\boldsymbol{m}_i + \boldsymbol{n}_i)$ and $\boldsymbol{S}_j = S(\boldsymbol{m}_j - \boldsymbol{n}_j)$. Regarding $\boldsymbol{n}_j = \boldsymbol{n}_i + a(\hat{\boldsymbol{r}}_{ij} \cdot \boldsymbol{\nabla})\boldsymbol{n}_i + \cdots$ and similar for $\boldsymbol{m}_j$, we obtain the continuum limit of Eq. (S1) as

$$H_D = \mathcal{D}[\boldsymbol{n} \cdot (\tilde{\boldsymbol{\nabla}} \times \boldsymbol{n}) + \tilde{\boldsymbol{\nabla}} \cdot (\boldsymbol{n} \times \boldsymbol{m}) - \boldsymbol{m} \cdot (\tilde{\boldsymbol{\nabla}} \times \boldsymbol{m})], \tag{S2}$$

where $\mathcal{D} = aS^2D$ with a the lattice constant, and $\tilde{\boldsymbol{\nabla}} = \hat{\boldsymbol{z}} \times \boldsymbol{\nabla}$. The second term of H_D is a total derivative that does not affect the local dynamics of the system. It worths emphasizing that if the i-sites are assumed to be B-sublattices (*i.e.*, interchange A and B sublattices), this total derivative term flips sign.

S2. SPIN WAVE SCATTERINGS ON A 1-DIMENSIONAL DMI WELL

In a system with translational symmetry in space and time, solutions to the wave equation take the form

$$\psi^{(k,\omega)} = e^{-i\omega t}(Ae^{ikx} + Be^{-ikx}), \tag{S3}$$

which we interpret as right- and left-moving spin waves in an easy-axis antiferromagnet (AFM). Now we consider three regions I, II, and III in a chain. Regions I and III are easy axis AFMs that extend to $\mp\infty$, while region II occupying interval $[0, L)$ is subject to an additional Dzyaloshinskii-Moriya interaction (DMI).

In region I, we have

$$\psi_I^{(\omega)} = e^{-i\omega t} \begin{pmatrix} Ae^{ikx} + Be^{-ikx} \\ \tilde{A}e^{ikx} + \tilde{B}e^{-ikx} \end{pmatrix}, \tag{S4}$$

the two components correspond to right (no tilde) and left (tilde) handed modes. Following Eq. (4) in the main text, the dispersion relation is (in the absence of the DMI)

$$k = \sqrt{\omega^2 - Z}, \tag{S5}$$

where we have set the spin wave velocity $c = 1$ for simplicity and Z is the (scaled) easy-axis anisotropy. We let $A = \tilde{A} = 1$, corresponding to a unit flux of both right- and left-handed modes, at frequency ω, incoming from $-\infty$. Due to *time* translational symmetry, a solution of the system will necessarily transform as $e^{-i\omega t}$. Therefore, a global frequency ω exists for the entire system. Let $k = k(\omega)$ be a local function for each region dictated by the locally satisfied dispersion relation.

For region II, the local solution is

$$\psi_{II}^{(\omega)} = e^{-i\omega t} \begin{pmatrix} Ce^{ipx} + De^{-ipx} \\ \tilde{C}e^{i\tilde{p}x} + \tilde{D}e^{-i\tilde{p}x} \end{pmatrix}. \tag{S6}$$

For region III, there are only transmitted waves, so

$$\psi_{III}^{(\omega)} = e^{-i\omega t} \begin{pmatrix} Ee^{ikx} \\ \tilde{E}e^{ikx} \end{pmatrix}. \tag{S7}$$

At $x = 0$ and $x = L$, the continuity of ψ and its x-derivative leads to

$$1 + B = C + D, \tag{S8}$$

$$ik(1 - B) = ip(C - D), \tag{S9}$$

$$Ce^{ipL} + De^{-ipL} = Ee^{ikL}, \tag{S10}$$

$$ip\left(Ce^{ipL} - De^{-ipL}\right) = ikEe^{ikL}. \tag{S11}$$

Similar equations for the left-handed branch can be derived by adding tildes in the obvious locations, so we will not write them explicitly. Let $\Delta_\pm = k \pm p$, the solutions are

$$B = \frac{\Delta_+\Delta_- \sin pL}{2ikp\cos pL + (k^2 + p^2)\sin pL}, \tag{S12}$$

$$C = \frac{2k\Delta_+}{\Delta_+^2 - e^{2ipL}\Delta_-^2}, \tag{S13}$$

$$D = \frac{-2k\Delta_-}{\Delta_+^2 - e^{2ipL}\Delta_-^2}, \tag{S14}$$

$$E = \frac{2ie^{-ikL}kp}{2ikp\cos pL + (k^2 + p^2)\sin pL}. \tag{S15}$$

In the limit that $p = k$ (no DMI), we have $B = D = 0$, *i.e.*, there is no reflection. In this case, the transmission is perfect ($A = C = E = 1$).

If the DMI is nonzero, however, we necessarily have reflection both at the gate entry and within the gate itself. Furthermore, since all the coefficients depend on p, the tilde'd coefficients for the left-handed branch are necessarily different from the right handed branch. Suppose that the length of the gate is chosen so that $pL = \frac{\pi}{2} + 2\pi N$ and $\tilde{p}L = -\frac{\pi}{2} + 2\pi N$, where N is an arbitrary integer. Then

$$E = \frac{2ie^{-ikL}kp}{(k^2 + p^2)}, \qquad \tilde{E} = -\frac{2ie^{-ikL}k\tilde{p}}{(k^2 + \tilde{p}^2)}. \tag{S16}$$

Following Eq. (4) of the main text, we can expand $p = (Q/2) + \sqrt{(Q/2)^2 + \omega^2 - Z}$ and $\tilde{p} = -Q/2 + \sqrt{(Q/2)^2 + \omega^2 - Z}$ to second order in $Q \sim D/J \ll 1$ and obtain

$$p = k + \frac{Q}{2} + \frac{Q^2}{8k} + O(Q^3), \qquad \tilde{p} = k - \frac{Q}{2} + \frac{Q^2}{8k} + O(Q^3). \tag{S17}$$

Using these expressions in E and $\tilde{E}$, we find that the ratio $E/\tilde{E} = 1 + O(Q^3)$. For reasonably weak DMI, then, the amplitudes of the left and right-handed modes in region III are equal to very good approximation.

Inside region II, assume again that $pL = \frac{\pi}{2} + 2\pi N$ and $\tilde{p}L = -\frac{\pi}{2} + 2\pi N$, we have

$$C = \frac{k\Delta_+}{k^2 + p^2}, \qquad \tilde{C} = \frac{k\tilde{\Delta}_+}{k^2 + \tilde{p}^2}, \tag{S18}$$

$$D = \frac{-k\Delta_-}{k^2 + p^2}, \qquad \tilde{D} = \frac{-k\tilde{\Delta}_-}{k^2 + \tilde{p}^2}, \tag{S19}$$

where $\tilde{\Delta}_\pm = k \pm \tilde{p}$. Unlike the ratio $E/\tilde{E}$, the ratios $C/\tilde{C}$ and $D/\tilde{D}$ differ at first order in Q (upon the second order expansion of $\tilde{p}$). Therefore, we expect to notice small elliptical openings (on the order of Q/k) of modes inside the gate.

S3. DETECTION OF SPIN WAVE STATE THROUGH SPIN PUMPING

The detection consists of two successive processes: spin pumping into the heavy metal by the precessing Nèel order, and inverse spin Hall effect that converts the pumped spin into a voltage.

The pumped spin accumulation is given by $\langle \delta \boldsymbol{s} \rangle \sim \boldsymbol{n} \times \dot{\boldsymbol{n}}$ [1], which relates to the inverse spin Hall voltage by $\boldsymbol{V} \sim \hat{\boldsymbol{y}} \times \langle \delta \boldsymbol{s} \rangle$ [2] based on the device geometry illustrated in Fig. 1. Two voltmeters V_1 and V_2 are used to measure the z- and x-component of the generated voltage [3]. Since only small oscillations of $\boldsymbol{n}$ around the $\hat{\boldsymbol{z}}$-axis concerns

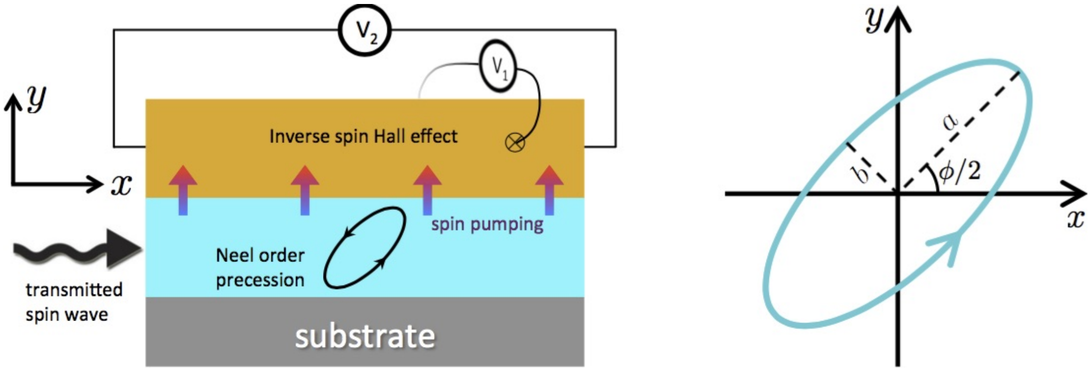

FIG. 1. Detection of spin wave transmitted from the gated region via spin pumping. Voltmeters V_1 (V_2) measures the inverse spin Hall voltage along z (x), corresponding to the pumped spin accumulation along x (z) that stems from the Nèel order oscillation along y (in the $x-y$ plane).

us, it is straightforward to see the following fact: the y-component of the oscillation is reflected by V_1 which is purely AC, whereas the area of the orbit in the $x-y$ plane gives rise to V_2 that involves both AC and DC components.

Now we determine a spin wave state specified by θ and ϕ in terms of the readouts V_1 and V_2. Under the basis of the circularly-polarized modes

$$|R\rangle = \begin{pmatrix}1\\0\end{pmatrix}, \quad |L\rangle = \begin{pmatrix}0\\1\end{pmatrix}, \tag{S20}$$

a spin wave state is expressed as

$$|\psi\rangle = \mathcal{C}\left[\cos\frac{\theta}{2}|R\rangle + \sin\frac{\theta}{2}e^{i\phi}|L\rangle\right], \tag{S21}$$

where $\mathcal{C}$ is the spin wave amplitude fixed by the source. For $\theta = 0$, $|\psi\rangle = \mathcal{C}|R\rangle$ is purely right-handed, the DC component of V_2 reaches its maximum $\bar{V}_2^m$. Here, we ignore the attenuation of spin waves across the gated region. Thus, by comparing the measured $\bar{V}_2$ with its maximum $\bar{V}_2^m$, we are able to determine θ. As shown in Fig. 1, the semi-major and semi-minor axes of the elliptical orbit is $a = \mathcal{C}(\sin\theta/2 + \cos\theta/2)$ and $b = \mathcal{C}(\sin\theta/2 - \cos\theta/2)$, respectively. So the area of the ellipse is $\pi ab = \mathcal{C}^2\cos\theta$. Since $\bar{V}_2$ is proportional to this area, the angle θ is then

$$\theta = \arccos\frac{\bar{V}_2}{\bar{V}_2^m}, \tag{S22}$$

which proves Eq. (9a) in the main text.

In the absence of the gate voltage, a y-polarized spin wave generated by the source is transmitted with its polarization unchanged. In this case, V_1 reaches its maximum V_1^m. Since V_1 is purely AC, we understand it here by its effective value $\tilde{V}_1$. For an arbitrary spin wave state labeled by (θ, ϕ), the measured $\tilde{V}_1$ is reduced to

$$\tilde{V}_1 = \tilde{V}_1^m\left[\cos\frac{\theta}{2} + \sin\frac{\theta}{2}\right]\sin\frac{\phi}{2}. \tag{S23}$$

By plugging in $\cos\theta = \bar{V}_2/\bar{V}_2^m$, we arrive at

$$\phi = 2\arcsin\left[\frac{\tilde{V}_1}{\sqrt{2}\tilde{V}_1^m}\frac{\bar{V}_2^m}{\bar{V}_2}\left(\sqrt{1+\frac{\bar{V}_2}{\bar{V}_2^m}} - \sqrt{1-\frac{\bar{V}_2}{\bar{V}_2^m}}\right)\right], \tag{S24}$$

which proves Eq. (9b) in the main text.

[1] R. Cheng, J. Xiao, Q. Niu, and A. Brataas, Phys. Rev. Lett. **113**, 057601 (2014).
[2] T. Kimura, T. Otani, T. Sato, S. Takahashi, S. Maekawa, Phys. Rev. Lett. **98**, 156601 (2007).
[3] H.-J. Jiao and G. E. W. Bauer, Phys. Rev. Lett. **110**, 217602 (2013).
